# Sclerosing mesenteritis and mesenteric panniculitis – clinical experience and radiological features

**DOI:** 10.1186/s12876-017-0632-7

**Published:** 2017-06-13

**Authors:** Lisa Nyberg, Jan Björk, Peter Björkdahl, Olle Ekberg, Klas Sjöberg, Lina Vigren

**Affiliations:** 1 Department of Medicine, Division of Gastroenterology, Hospital of Trelleborg, Trelleborg, Sweden; 2 Department of Gastroenterology and Hepatology, Karolinska University Hospital, Stockholm, Sweden; 3 Department of Radiology, Division of Surgery, Ystad Hospital, Ystad, Sweden; 4 Department of Translational Medicine, Division of Medical Radiology, Skåne University Hospital, Lund University, Malmö, Sweden; 5 Department of Clinical Sciences, Division of Gastroenterology, Skåne University Hospital, Lund University, Malmö, Sweden

**Keywords:** Clinical classification, Mesenteric panniculitis, Mesenteritis, Radiological classification, Panniculitis, Retractile mesenteritis, Sclerosing mesenteritis

## Abstract

**Background:**

Sclerosing mesenteritis (SM) is sometimes used as an umbrella-term for idiopathic inflammatory conditions in the mesentery. Mesenteric panniculitis (MP) is a radiological finding and its relation to clinical SM is not fully understood. The aims of this study were to determine whether any correlation could be found between the radiological findings and the clinical disease course.

**Methods:**

Patients observed due to idiopathic inflammation of the mesentery were identified. If SM could be verified histologically or MP radiologically, the patients were included in this descriptive retro perspective study.

**Results:**

Typical radiological changes were observed in 27 patients. A majority (23/27) of these patients had mild to moderate symptoms. This group with typical radiology was labelled MP. Four patients were included due to histologically verified disease but had uncharacteristic radiology involving multiple compartments of the abdomen. All four had marked systemic inflammation, fever and fluctuating radiologic findings. Three had severe disease with multiple hospitalisations and complications but responded promptly to corticosteroids. This group was denoted SM.

**Conclusions:**

We have identified two subgroups of patients; firstly, MP with stable and characteristic radiologic changes and secondly SM with atypical radiology and a more aggressive clinical course. We propose that the term SM should be reserved for this latter condition.

## Background

Sclerosing mesenteritis (SM) is a rare but probably underdiagnosed condition of inflammation in the mesentery. It was first described in 1924 under the term “retractile mesenteritis” and over the years, mesenteric panniculitis (MP), and mesenteric lipodystrophy [1, 2] have also been used to describe similar conditions of inflammation in the mesentery. A histological study by Emory et al. suggested the use of SM as an umbrella term since they histologically seemed to be one entity and only represented different stages of the same disease [3]. The umbrella term SM has been widely accepted in clinical studies although it is yet to be clarified if these histologically similar conditions share clinical and radiological features.

The diagnosis is based on histopathology or radiology. Histology was the most reliable diagnostic tool earlier and was considered as the standard for diagnosis and has been used in some major clinical studies [4]. Radiology is the most accessible diagnostic modality today and many recent studies have used radiological criteria. The term commonly used in radiological studies is MP [5–8]. Typical findings on computer tomography (CT) are a solid fatty mass in the mesentery of the jejunum with lymph nodes and a pseudo capsule surrounding the lesion [5, 6, 8]. Histopathology usually shows fat necrosis, fibrosis and some degree of chronic inflammation with lymphocyte infiltration [3, 9]. The typical radiological findings are often referred to in clinical studies but major clinical SM studies [4, 10] have not evaluated if the typical radiological findings correlate to the severity of the disease. The suggestion by Emory et al. [3] that MP is a subgroup of SM has not been questioned. A clinical study based on radiological findings by Van Putte et al. [8] has however proposed that MP should be separated from retractile mesenteritis as the MP changes did not progress into mesenteric fibrosis.

The reported prevalence in radiological studies ranges from 0.6 to 2.5% [1, 8, 11]. The prevalence in clinical practice has not been studied but as an example only 92 cases were identified over a period of 23 years at the Mayo clinic, Rochester, which suggests a discrepancy between clinically relevant cases and the radiological finding in MP. The condition has earlier been described as benign but the clinical course may vary from no symptoms to severe and aggressive disease [4]. When symptoms are present they are usually unspecific, such as abdominal pain, nausea, fever and weight loss [4]. Blood biochemistry is often normal but occasionally elevated CRP, mild anaemia and hypoalbuminemia can be present [8, 10].

Inflammation in the mesentery can also occur as a secondary phenomenon due to several different conditions. The differential diagnoses comprise other local inflammatory processes or neoplasms [12]. In some studies it has been suggested an overrepresentation of SM in patients with malignant disease [1, 7, 8], although a matched pair analysis has questioned this [6].

The first line treatment of SM is corticosteroids but sometimes other immune modulating agents such as Thiopurines [10, 13, 14] and TNF inhibitors [15] have been tried as well as colchicine [16] and thalidomide [17]. Hormone therapy with tamoxifen has also been used [4].

The aims of this study were to summarise the clinical experience from two regions in Sweden, to determine whether any correlation could be found between the radiological findings and the clinical disease course and to suggest an appropriate follow up strategy.

## Methods

Gastroenterologists and surgeons in the county of Skåne were requested to report all cases with SM/MP. A registry with SM/MP patients was already set up in Stockholm by a local expert in the field (JB). From both regions, known patients with clinical SM diagnosed 2005–2014 were collected. In the county of Skåne, the in-patient registry was used for identification of the patients (based on the ICD-10 code K668, other specified diseases in the peritoneum). A letter with information about the study was sent to all patients giving them the possibility to decline participation (the opt-out principle). The participants’ medical records were collected form each hospital. Data on disease history, laboratory tests and histopathology were collected. Results of CT examinations were collected.

### Diagnostic criteria

**Fig. 1:**
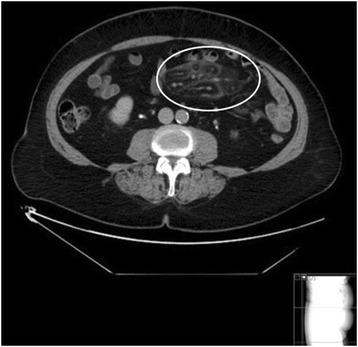
Moderate radiological SM with a well-defined fatty mass in the jejunal mesentery without mass effect (1p), hyperattenuation of the fat (3p), lympnodes (2p), halo (2p) and a pseudocapsule (1p)

**Fig. 2:**
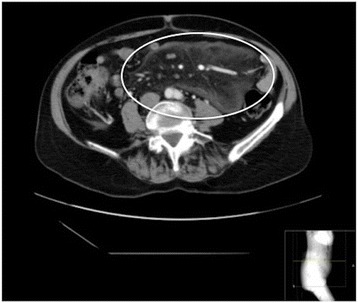
Extensive radiological SM with a large well defined fatty mass in the small intestine mesenteria (3p), marked hyperattenuation of the fat (3p), multiple lymphnodes (3p) with halo (3p) and a clear pseudocapsule (3p)

The medical records were reviewed and if the diagnosis could be confirmed with histology or radiology, the patients were included. The patients with typical radiological appearance on CT were labelled MP and the histologically confirmed cases with atypical radiology were labelled SM. Sclerosing mesenteritis was considered histologically confirmed if the pathologist suggested SM or if the clinician concluded the inflammatory changes consistent with SM. A group consisting of two radiologists and two clinicians reviewed the CT examinations. The Coulier CT criteria were used for radiologic inclusion. [4, 5] Mesenteric panniculitis was considered confirmed if three out of five criteria were present: (A) Fatty mass lesion in the small intestinal mesentery, (B) hyper attenuation of the fat, (C) lymph nodes in the fatty mass, (D) halo surrounding lymph nodes or vessels and (E) pseudo capsule. The images were graded using a scoring system based on the five diagnostic criteria (A-E). Scores 0–3 were given for each criterion. Zero corresponded to no pathological findings and 3 to extensive findings. A total score of 3–4 represented mild, 5–10 moderate and 11–20 extensive radiological changes. Examples can be seen in Figs. 1 and 2.

If the observed changes could be explained by adjacent pathology such as a neoplasm or other defined inflammation in the area (secondary mesenteritis) the patient was excluded.

Since the criteria for establishing the diagnosis based on findings on Magnetic Resonance Imaging (MRI) alone are not yet defined, patients exclusively examined with MRI were not included in this study.

### Clinical scores

Medical records were used to grade the severity of the symptoms. The patients were divided into four different categories: Asymptomatic patients (score 1), symptomatic but without systemic signs of inflammation (normal CRP and no history of fever) (score 2), symptomatic with systemic signs of inflammation (elevated CRP and/or fever due to SM were no other apparent explanation could be found) (score 3) and severe disease (chronic disease, complications, multiple hospitalisations or therapy resistant disease) (score 4).

### Statistics

Putative correlations were estimated with Spearman’s non-parametrical test. SPSS version 22 was used for calculations. A *p*-value below 0.05 was considered significant. Data are presented as median and interquartile.

## Results

In total 36 patients diagnosed 2005–2014 were identified. None declined participation. Five patients could not be included. One had secondary mesenteritis due to vasculitis and the other two had too discrete radiological changes to fulfil the radiological criteria and specimens for histological examination were absent. Moreover, two patients were only examined with MRI.

Radiological criteria were fulfilled in 27 patients (MP) and three of them also had a histological confirmation. Four patients had a histological diagnosis but did not meet the radiological criteria (SM). These two subgroups will be presented separately.

### Mesenteric panniculitis

Median age at diagnosis was 50 (IQR 44; 72) years. Eleven patients were women and 16 were men. Median age at diagnosis was 64 years in women and 46 years in men. The male patients were diagnosed significantly earlier than the female patients (*p* = 0.038). Six of the 27 patients had autoimmune diseases (psoriasis arthritis and sarcoidosis in one patient and psoriasis, Bechet’s disease, coeliac disease, hypothyreosis and Crohn’s disease), respectively, in the others).

### Symptoms

Five of the 27 patients were asymptomatic, 13 had symptoms without signs of systemic inflammation, five were symptomatic with signs of systemic inflammation and four had severe disease with multiple hospitalisations, chronic, refractory or complicated disease. All four of the patients with high clinical score had a concomitant chronic disease (Bechet’s, Crohn’s, psoriasis arthritis and hereditary spastic paraparesis) and for three of them, the concomitant disease caused the major morbidity. All nine patients with clinical score three and four had elevated CRP at diagnosis and two had fever. None of the 27 patients increased their clinical score, had severe complications of their SM or died during follow-up.

Abdominal pain was reported by 21 patients and was the most common symptom. Six patients specifically reported symptoms at night and symptoms related to body posture. In addition, nausea, weight loss, flatulence and diarrhoea were reported in occasional patients. Tenderness in the left hypochondrium and sometimes a tender palpable mass was described. Most symptomatic patients had chronic discomfort but some patients had acute episodes with intense pain, sometimes with mild to moderately elevated CRP levels. No correlation was seen between the radiological score and the clinical score (*p* = 0.68), nor was any correlation seen between clinical score and age or gender (*p* = 0.16 and 0.62, respectively).

### Treatment

Eight patients were treated with anti-inflammatory agents for active MP. Three underwent diagnostic surgery; none had surgery with intention to treat. All eight patients with anti-inflammatory treatment were initially given corticosteroids. They were given an initial dose of 20–40 mg prednisolone and tapering was usually made over 8–12 weeks. Six patients responded and two patients had none or poor response to corticosteroids.

Four patients were treated with other immunomodulating agents during follow-up. All of these patients had also reported an initial response to corticosteroids. Three were treated with thiopurines. One responded well and needed no other treatment. One responded partially and one had to stop medication due to side effects.

One patient was treated with a TNF inhibitor. He was under medication with adalimumab (Humira®) and methotrexate due to psoriasis arthritis when he was diagnosed with SM. The adalimumab treatment was discontinued and 40 mg prednisolone was initiated causing reduction of symptoms. Later etanercept (Enbrel®) was initiated as treatment of the psoriasis arthritis leading to decrease of abdominal pain.

### Radiology

Of the 27 patients who fulfilled the CT criteria two had mild radiological changes, 21 had moderate and four had extensive radiological changes. Mean score was 8 (range 4–15). The severity of the radiological changes did not correlate with age or gender (*p* = 0.68 and 0.94, respectively). The most common findings were a well-defined fatty mass in the mesentery of the small intestine and hyper attenuation of the fat. All patients had these changes in at least one CT examination. Lymph nodes in the fatty mass were found in 26/27. As for the more SM specific changes: i.e. halos surrounding lymph nodes or vessels and pseudo capsule, at least one of these signs occurred in 23/27. Halos surrounding lymph nodes or vessels could be observed in 16/27 and 16/27 had a pseudo capsule. None of the patients had ascites.

Repeated CT scans were carried out in 19 patients. These patients had a median radiological observation time of 37 months (IQR 9;50). Six patients had a radiological regress, eight a radiological progress and six patients had no change in score. There was no significant progress of the radiological changes (mean 0, range − 4 to 3) and the mean variability from diagnostic CT to the last one was 1 point during the observation time.

### Sclerosing Mesenteritis

A subgroup of four patients with mesenteric inflammation and histologically verified SM were identified. All four of them had clinical symptoms and findings as well as histological features compatible with SM. Although they had extensive radiological findings they did not fulfill the Coulier criteria for MP. The term SM was reserved for these patients.

#### Symptoms

Three out of four patients had severe disease with clinical score four and one had clinical score three. One patient died during follow-up due to complications from her SM. Two patients had bilateral hydro nephrosis and retroperitoneal fibrosis in addition to the mesenteric changes. One patient also had relapsing small bowel obstruction, colon obstruction and intestinal strictures. All patients had abdominal pain, fever, small amounts of ascites and elevated CRP. Moreover, all four had a relapsing remitting disease course. All had diagnostic surgery and two had repeated surgery due to complications.

#### Radiology

All four patients had radiological changes in more than one compartment of the abdomen (retroperitoneum, peritoneum viscerale, peritoneum parietale or omentum). The changes were less well defined than the MP changes and lacked distinct demarcation towards surrounding tissue present in MP, often even as a pseudopapsule. Lymph nodes were seen but not surrounded with a halo. The changes were highly fluctuating in all cases, changing in extent and sometimes localization and in two cases they resolved almost completely (spontaneously in one case and after corticosteroid treatment in another). See Table 1.

**Table 1:** Summary of the SM group

Gender Age	Clinical score	Complication	CRP mg/L	Alb. g/L	Platelets ×10´9/L	Temp C	Hb g/L	Treatment	Ascites
F 63	4	Bilateral hydronefros	90	23	508	38,0	85	Prednisolone: immediate response	Yes
F 43	4	Colonobstruction, hydronefrosis	259	16	784	38,2	86	Prednisolone: immediate response. Eventually stabilised on azatihoprine and adalimumab.	Yes
M 29	3	0	5	39	268	40,0	135	None	Yes
F^a^ 71	4	Mors^a^	246	24	243	38,3	117	Prednisolone: immediate response, Azatioprin, Tamoxifen, Adalimumab, Infliximab tried (see case report)	Yes

Values measured during symptomatic flare

^a^See Case report

#### Treatment

Corticosteroids were initiated in three out of four patients. In these three cases, the treatment was effective on abdominal pain, biomarkers and radiological changes, usually within days. Two patients needed long term immunomodulating treatments during follow-up. For the third patient who later died, immunomodulatory was initially very effective but after a couple of years, higher doses were needed (see case presentation).

##### Case presentation from the SM group in this study

**Fig. 3:**
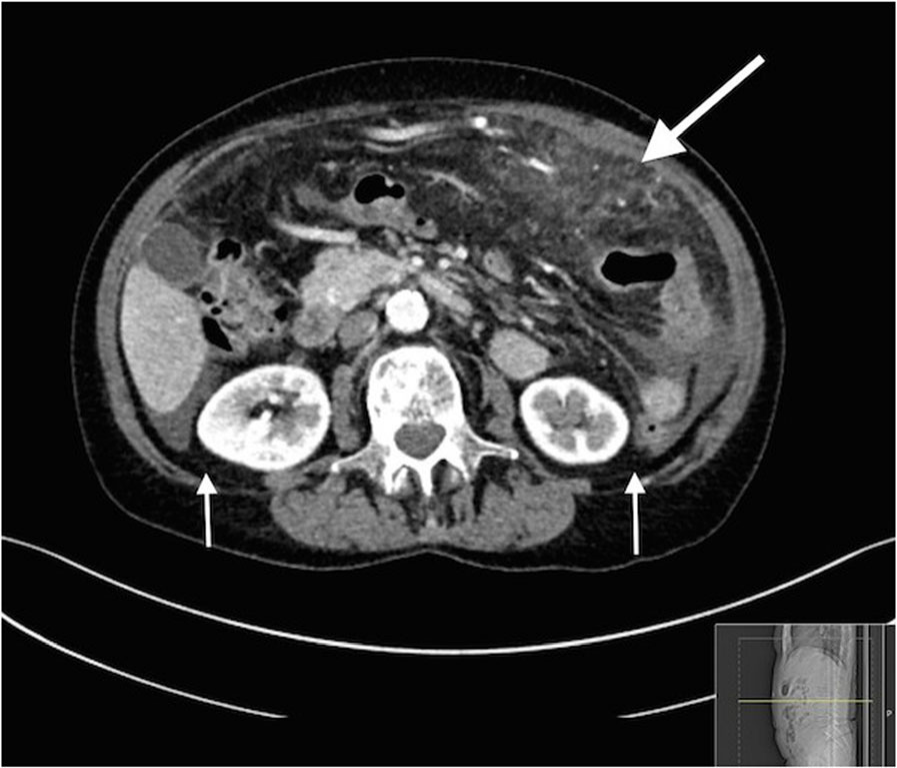
A 71 year old female with sclerosing mesenteritis. (Presented as case) There is diffuse increased density in the small bowel mesentery anteriorly in the upper abdomen (*arrow*). There is also involvement of the greater omentum. No capsule or enlarged lymphnodes are present. Small amounts of ascites is seen in the lateral colonic gutters (*small arrows*, Fig. 3) In the small pelvis (Fig. 4) there is increased density in the mesentery to the sigmoid colon (*arrow*)

**Fig. 4:**
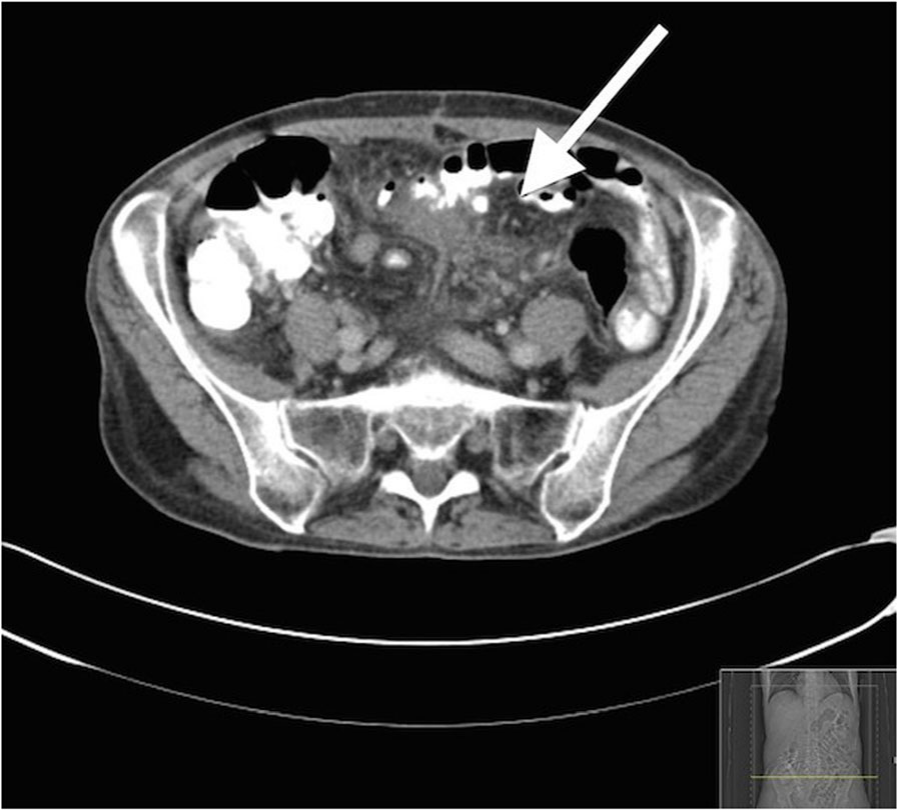
A 71 year old female with sclerosing mesenteritis. (Presented as case) There is diffuse increased density in the small bowel mesentery anteriorly in the upper abdomen (*arrow*). There is also involvement of the greater omentum. No capsule or enlarged lymphnodes are present. Small amounts of ascites is seen in the lateral colonic gutters (*small arrows*, Fig. 3) In the small pelvis (Fig. 4) there is increased density in the mesentery to the sigmoid colon (*arrow*)

A 71-year-old female presented with abdominal pain. CT showed porta vein thrombosis, ascites and inflammation around the appendix. She was treated conservatively for suspected appendicitis. Over the following years, she relapsed with acute severe abdominal pain, fever, dramatically elevated CRP, anemia and hypoalbuminemia. Repeated CT scans were performed showing various grades of inflammation on different locations in the omentum, and mesentery (Figs. 3 and 4). She was examined for infectious and malignant causes and even a laparotomy was performed and histopathological analysis of inflamed omentum showed unspecific inflammation, mesotel cell proliferation, histiocytes and fibrosis. Prednisolone with an initial dose of 20–40 mg in tapering doses was initiated with initially good effect. Due to frequent relapsing symptoms, higher prednisolone doses were required, usually with a prompt clinical, laboratory and radiological response. After 4 years, the disease was refractory and 60 mg prednisolone was given when relapsing. Tamoxifen was tried but she did not respond. Adalimumab and infliximab were also tried but were discontinued due to lack of effect and intolerance, respectively. Six years after onset, the patient developed end stage disease and finally died at a palliative unit. Her severe pain was treated with high doses transdermal Fentanyl and she had a continuous prednisolone dose of 60 mg daily.

## Discussion

When studying the literature on this subject it becomes clear that the terms used describing primary inflammatory conditions in the mesentery are inadequately defined and inconsistent. Early on, patients with abdominal symptoms and typical histopathology were diagnosed as SM. With increasing access to CT, radiology has to some extent replaced biopsy as a diagnostic tool. MP is a well-defined entity radiologically (well-defined hyperdens fatty mass, lymphnodes with halos surrounded by a pseudo capsule) but sometimes clinically used synonymously with SM. This is probably a consequence of the unspecific histopathology present in both SM and MP, which has led to vast confusion. To the best of our knowledge, no study has yet confirmed if the radiological changes in MP correlate with the severity of disease. This study is a descriptive, retro perspective study and the results need to be confirmed. However, we found that typical radiological changes of MP, regardless of symptoms and histopathology, usually correspond to a stable disease with little risk of progression or complications. Consequently, in this study we have been able to identify a subgroup of patients with MP that have a much more benign disease course using radiological criteria.

In the present study, many patients with very mild or no symptoms that could be related to MP had undergone repeated CT: s because of the extensive radiological findings. In one of our asymptomatic patients, five follow-up CT:s were carried out over a period of 7 years. These investigations have generated approximately 60 mSv and this alone corresponds to 20 years of background radiation [18]. The possibility of MP being a para-malignant phenomenon may contribute to this, but also fear of progressive disease. The possible connection between MP and malignancy should motivate a thorough clinical history and clinical examination at diagnosis but does not motivate repeated radiological examinations if the symptoms are stationary. Since peritoneal mesotelioma or lymphoma can also present itself in similar ways, atypical changes or changes that are associated with alarm symptoms may require biopsy. The radiological changes seen on CT did not disappear in any of our patients and seldom varied during follow-up. This has also been shown earlier in radiological studies based on CT criteria [1, 8, 11]. This study has demonstrated that the patients with radiologically typical MP according to the Coulier criteria [5] mostly have a stable and usually mild to moderate disease. We suggest that this subgroup should be called MP. Taken this into account, we do not consider repeated radiological examinations to be an appropriate method for evaluation of the disease severity over time since the diagnosis has been established. Nor does it seem to be an appropriate method to evaluate the effect of treatment or even the patient’s need for treatment. Consequently, decisions about the follow-up of MP patients should primarily be based on the clinical picture, something that also has been concluded previously [4].

The most common symptom in our MP is abdominal pain, often accentuated at night and related to body posture. Even though the disease course is mostly benign occasional patients may need treatment. Most patients (6/8 in the present study) have been treated with corticosteroids (prednisolone) and they responded clinically with improvement of the symptomatology. Corticosteroids seem to be effective and recommended as first line of treatment. Thus, in all symptomatic cases with MP, regardless of CRP level, corticosteroids could be tried. We recommend prednisolone 40 mg and effect evaluation within 2 weeks. If effective, tapering off with 5 mg per week is appropriate, slower below 10 mg. If a distinctive clinical and/or biochemical response is evident on corticosteroids, immunomodulating treatment with thiopurines for long-term use could be considered. Surgery seems to be of little value in the treatment of MP as the inflammatory mass involves vessels to the small intestine. It has previously been stated that surgery should be limited to treating severe complications [19].

In the literature, SM is sometimes presented as a relatively common condition with a benign disease course. However, numerous case reports indicate that the disease course can be severe and even lethal. Our data indicates that unspecific radiological changes and histopathological changes that are interpreted as SM may correspond to a more aggressive clinical course with a high risk of complications and even death. In patients with marked unclear mesenteric changes that do not fulfill the radiological criteria for MP malignancy is usually suspected. Histopathology is necessary to differentiate between malignancy and an inflammatory process and usually, investigations to rule out an infectious cause is required. We have chosen to specifically denominate this group “sclerosing mesenteritis”. Although the SM and MP appear to have histological similarities shown by Emory et al. [3] the clinical and radiological differences shown in this study support that they may be different entities. The SM patients had more extensive inflammation that involved extra intestinal tissue in multiple compartments in the abdomen and had a radiological appearance different from that of MP. The radiological changes seen in the SM group did not at any point of the disease course resemble MP and presented with a pronounced fluctuation that could not be observed in MP. They all had aggressive disease with laboratory and clinical signs of extensive systemic inflammation, whereas the MP group usually had normal and in few cases mild systemic inflammation. SM also had a better response to corticosteroid treatment with prompt clinical, laboratory and radiological improvement. In our study, no conclusions can be drawn considering immunomodulation treatment in the SM group. Further studies are needed although the impression from our cases is that Azathioprine and anti TNF can be of value. CRP has been suggested as a pseudomarker for therapy response [17]. In line with our observations many of the complicated cases reported in the literature do not have the characteristic radiological findings of MP and share features with our SM group. This is the first study that has identified a subgroup with localised disease (MP) that can be separated from the patients that are more likely to suffer from complications and multifocal sclerosis (SM).

## Conclusions

MP with typical radiological findings is usually clinically and radiologically stable over time with mostly none or mild symptoms. Radiology is useful for diagnosis but the correlation between the radiological score and the clinical severity is poor and radiological progress over time seems uncommon. Therefore, radiology is not needed for routine follow-up in typical cases when malignancy has been ruled out. Corticosteroids are first line treatment and thiopurines may be useful if maintenance treatment is needed. Atypical radiology with histopathology compatible with SM may represent a separate entity with a more aggressive clinical course. This group has fluctuating radiological changes in multiple compartments of the abdomen but a prompt response to corticosteroid treatment. We propose that the term SM should be reserved for this condition.

## Abbreviations

Alb: Albumin
CRP: C Reactive Protein
CT: Computer tomography
F: Female
Hb: Hemoglobin
IQR: Interquartile range
M: Male
MP: Mesenteric panniculitis
MRI: Magnetic resonance imaging
mSv: Milli Sievert
SM: Sclerosing Mesenteritis
TNF: Tumour necrosis factor
